# Novel non-covalent stable supramolecular ternary system comprising of cyclodextrin and branched polyethylenimine

**DOI:** 10.1007/s10847-016-0677-1

**Published:** 2016-11-09

**Authors:** Artur Kasprzak, Magdalena Poplawska, Hanna Krawczyk, Sergey Molchanov, Mikolaj Kozlowski, Michal Bystrzejewski

**Affiliations:** 1 Faculty of Chemistry, Warsaw University of Technology, 00-664 Warsaw, Poland; 2 Department of Chemistry, University of Warsaw, 02-093 Warsaw, Poland

**Keywords:** Cyclodextrin, Polyethylenimine, Supramolecular chemistry, Spectroscopy

## Abstract

**Abstract:**

The synthesis of a novel supramolecular system comprising of branched polyethylenimine and cyclodextrin, is presented. The synthesis route is based on the self-assembly phenomena with the inclusion of solvent molecules. The systems are formed by a hydrogen-bonding network and host–guest type interactions between the building blocks. It was found that the native cyclodextrin and polyethylenimine are able to form stable systems when the reaction medium constitutes a polar solvent forming host–guest type complexes with cyclodextrin. A special consideration was paid on the detailed spectroscopic analyses of the obtained water-soluble constructs, including ROESY and diffusion-ordered (DOSY) NMR spectroscopy studies. The versatility and significance of DOSY technique for the analysis of the cyclodextrin complexes and its non-covalent systems with branched polymers, were presented. It was also found that the guest molecules that were incorporated in the complexes exhibited enhanced thermal stability. The morphological details in the solid state were obtained by scanning electron microscope.

**Graphical Abstract:**

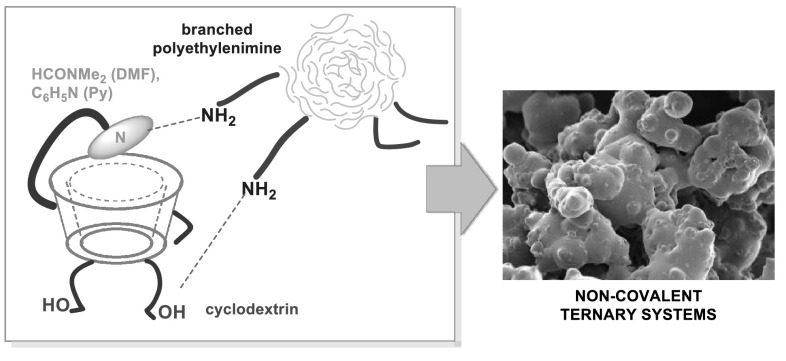

**Electronic supplementary material:**

The online version of this article (doi:10.1007/s10847-016-0677-1) contains supplementary material, which is available to authorized users.

## Introduction

Cyclodextrins (CDs) are a class of cyclic compounds, comprised of six or more repeating d-glucose units which are coupled by the α-1,4-glycosidic bond [1, 2]. The cyclodextrins chemistry is the so-called host–guest chemistry due to their original and interesting three-dimensional structure based on forming of the cup-shape supramolecules [3, 4]. The stereochemical arrangement of the cyclodextrin ‘cup’ implies that the exterior of CD is more hydrophilic in comparison with its interior. Therefore, the CD molecule is capable to bind the hydrophobic molecules in aqueous media, in order to increase their water solubility. The examples of a smart application of the aforementioned host–guest phenomena are (1) the anti-corrosion application (corrosion inhibition) [5], (2) the enhancing oil recovery [6], (3) as well as the complexation of anticancer drugs inside CD interior [7, 8]. A large number of drugs have poor water solubility, therefore the creation of CD-drug complexes enables (1) to reduce the required pharmacological effective dose of the anticancer drug and (2) to enhance its pharmacological availability. The aqueous stability of CD-drug inclusion complexes determines the possibility for designing the so called controlled drug release systems [9].

The unique properties of cyclodextrins resulted in many applications e.g. in the polymer science (synthesis of CD-polymer conjugates) [10–12] or sensors technology [13]. The CDs may constitute as the building blocks of the so-called self-assembly structures, which may be regarded as the promising starting-point for the synthesis of advanced materials [14]. An interesting method is to create the redox-responsive and shape memory polymer structures, based on the presence of CD covalently conjugated to a (bio)macromolecule [15]. Also, it was found that on the basis of host–guest interactions, the CD is not only able to complex simple molecules (like the aromatic compounds [16]. or typical organic solvents [17]), but also linear polymers, as for example poly(ethylene glycol) (PEG) [18] or poly(acrylamide) [19] Interestingly, under appropriate pH conditions the CD is also capable to interact with typical hydrophilic linear macromolecules, i.e. the linear polyethylenimine (PEI) [20] or PEG [21] part of the PEG-PEI copolymer, which results in formation of polyrotaxane-like structures.

Due to the interesting features of polyethylenimine, e.g. exhibited proton sponge effect or adsorption of various type of negatively charged chemical individuals, in the development of adsorption technologies the cyclodextrin-PEI (CD-PEI) *covalent* conjugates have been widely synthesized [22, 23]. These conjugation routes involve the use of sophisticated coupling reagents, e.g. 1,1′-carbodiimidazole (CDI) [24] or pre-modified CDs, e.g. tosylated (OTs-CD) cyclodextrin [25] or the hydroxypropylated one (HP-CD) [26]. Such covalent PEI-CD structures are considered as promising materials dedicated to bio-related field of application, including non-viral gene delivery systems. The PEI-CD covalent structures which form nanospheres in the way of self-assembly phenomena, with the inclusion of appropriate guest molecules, have been also reported [27]. Please note that most commonly the synthesis of such covalent type CD-polymer structures is associated with multistep conjugation protocol. Additionally, the cyclodextrin pre-derivatization process is not always connected with the satisfactory yield of the desired product. Therefore, the approach for the formation of polymer-CD systems based on the direct protocol (without pre-derivatization of the starting reactants) may constitute a promising and interesting proposal of the high-yield synthesis.

**Fig. 1:**
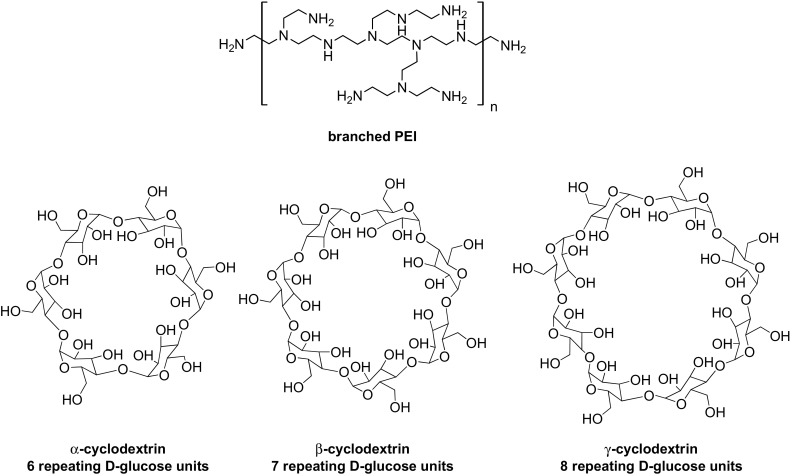
Chemical structures of branched PEI (*top*) and cyclodextrins (*bottom*)

Please note that a significant number of the synthetic protocols for conjugation of CD to branched PEI, is conducted (1) in DMF as a reaction medium (e.g. CDI-mediated approach [24]) or (2) with addition of pyridine (e.g. CD tosylation [25]). Nevertheless, there are no studies on the possible creation of the non-covalent PEI-CD supramolecular systems, despite of the fact that both PEI and CD is soluble in DMF and pyridine. In other words, a possible formation of PEI/CD non-covalent systems with the inclusion of DMF or pyridine molecules, has not been investigated yet. Herein we report a novel ternary *non-covalent* supramolecular system comprising the branched PEI (M_w_ = 25 kDa) and cyclodextrin (α, β or γ). The chemical structures of the used cyclodextrins and branched PEI are presented in Fig. 1. We have developed a novel and efficient approach to synthesize PEI aggregates bearing the CD and appropriate solvent molecules (DMF or Py), on the basis of the hydrogen-bonding interactions and host–guest phenomena. In this work, we present the in-depth insight into the spectroscopic studies of PEI-CD-guest systems both in aqueous media and solid state in order to increase the understanding of such strong non-covalent interactions.

## Experimental section

### Materials and methods

Branched polyethylenimine (PEI; >99%; M_w_ (by LS): 25 kDa) and cyclodextrins (αCD, γCD > 98%; βCD > 95%) were purchased from Sigma-Aldrich. Pyridine (Py; >99%) and *N*,*N*-dimethylformamide (DMF; >99.8) were purchased from Avantor Performance Materials Poland S.A. All the reagents were used as received without purification.

Fourier transformation infrared (FT-IR) spectra were recorded in a transmission mode with Thermo Scientific Nicolet iS5 spectrometer with a resolution of 4 cm^−1^. The samples were analyzed as pellets with dry KBr, whilst PEI was applied as thin film onto a pellet made of spectrally pure KBr. All NMR experiments were carried out on a Varian VNMRS spectrometer operating at 500 MHz and equipped with a multinuclear z-gradient inverse probehead. In all experiments, the probe temperature was maintained at 298 K and standard 5 mm NMR tubes were used. ^1^H NMR and ^13^C NMR spectra were recorded in deuterium oxide (with the calibration on the residual HOD signal 4.79 ppm and the cyclodextrin’s C1 signal (αCD—101.29 ppm, βCD—101.75 ppm, γCD—101.55 ppm), respectively). No internal reference was added, in order to eliminate possible interactions with CDs, if any. MestRe-C 2.0 software was used for NMR spectra simulation (*MestRe*-*C NMR Data Processing Made Easy 4.9.9.6, 1996*–*2006, courtesy F.J. Sardina, Universidad de Santiago de Compostela, Spain*).

DOSY (Diffusion Ordered SpectroscopY) experiments were performed using a stimulated echo sequence incorporating bipolar gradient pulses [28] and with convection compensation [29]. The gradient strength was logarithmically incremented in 15 steps from 25% up to 95% of the maximum gradient strength. The DOSY Toolbox software was used for DOSY NMR spectra simulation (*The DOSY Toolbox*—*version 2.5, 2014, Mathias Nilsson, School of Chemistry, University of Manchester, UK*).

Thermogravimetric analysis (TGA) was performed with a TA Q-50 instrument under nitrogen atmosphere and heating rate of 10 °C min^−1^.

Dynamic light scattering (DLS) measurements were performed using Malvern Zetasizer instrument. The analyses were conducted on the samples suspended in distilled water (100 μg mL^−1^).

Morphological features were obtained using scanning electron microscopy (Zeiss Merlin). The powdered samples were put on an aluminum holder coated with a carbon tape. Before observations the samples were covered with a thin layer of carbon using a commercial sputtering instrument.

### General procedure for the synthesis of the βCD-solvent inclusion complexes

A solution of the βCD (40 mg mL^−1^) in the appropriate solvent (DMF or Py) was stirred for 24 h at room temperature. The solvent was evaporated under reduced pressure. Finally, the residue was lyophilized for 24 h. The mass gain of the obtained products is: ca. 6% for DMF as the solvent and ca. 8% for Py as the solvent, of the mass of the βCD used for the reaction.

### General procedure for the synthesis of the PEI-CD-solvent ternary systems

The polyethylenimine (PEI; M_w_ = 25 kDa) was dissolved in the appropriate solvent (40 mg mL^−1^, *N*,*N*-dimethylformamide (DMF) or pyridine (Py)). To the stirred PEI solution, β-cyclodextrin dissolved in the same solvent (50 mg mL^−1^) was added dropwise. The PEI:CD mass ratio was 1:1. A turbid-white mixture was obtained and the formation of the aggregates was observed immediately. Despite the fact that the process is very rapid (please see Supplementary Video Data), the mixture was stirred overnight at room temperature to ensure the best reaction yield. Sequentially, the as-obtained system was centrifuged five times with the solvent used for the reaction) (DMF or Py) and the supernatant was removed. The content of unbonded cyclodextrin in the following supernatants was monitored by TLC (a phosphomolybdic acid test was used: a drop of a supernatant was placed on a TLC plate and phosphomolybdic acid was used to visualize CD). Finally, the product was lyophilized for 24 h. The masses of the obtained materials are summarized in Table 1. Hereafter, the obtained supramolecular structures are referred as PEI-CD-DMF and PEI-CD-Py, for the systems consisting *N*,*N*-dimethylformamide (DMF) and pyridine (Py), respectively.

**Table 1:** Experimental data for the synthesis of the PEI-βCD-solvent non-covalent supramolecular systems

Substrates			Products			
PEI mass (mg)	βCD mass (mg)	Solvent	Ternary system	Ternary system mass (mg)	Weight gain in comparison to PEI (%)	Weight gain in comparison to βCD (%)
204.8	201.2	DMF	PEI- βCD-DMF	317.6	55	58
198.7	197.5	Py	PEI- βCD-Py	336.2	69	70

Two representative reactions with αCD and γCD were also carried out applying DMF as the solvent in both cases. There were no significant differences in the course of the reaction, the final product yield and the aggregates morphology.

## Results and discussion

The synthesis of the ternary systems involves the addition of the CD solution in the appropriate solvent (DMF or Py) into the stirred PEI 25 kDa solution in the same solvent (PEI:CD mass ratio is 1:1). The process is very rapid, the aggregates are formed instantly and the first precipitate is observed even after few seconds (please see Supplementary Video Data). The obtained supramolecular systems exhibit interesting solubility behavior in the different media (Table S1 in Supplementary Data). Please note that the solubility behavior of the CD-solvent inclusion complexes is the same as for the native CD. Interestingly, despite of the fact that the constructs main building elements are the PEI and CD, the obtained supramolecular aggregates are not soluble neither in methanol or chloroform (PEI) nor DMSO (CD). If so, one has to consider that the strength of the hydrogen bonds between the obtained aggregates and solvent molecules is the main factor which crucially influences the solubility behavior.

Both cyclodextrin and polyethylenimine can act as the hydrogen bond (HB) donors or acceptors, due to the presence of specific functional groups, i.e. hydroxyl (CD) and amino (PEI) moieties, in their structure. Also, it was shown that each structure comprising of cyclodextrin is being stabilized by the host–guest type interactions [19]. Thus, the products were analyzed by means of spectroscopic methods in order to observe and to determine the supramolecular interactions (if any) in the obtained non-covalent systems.

## ^1^H and ^13^C NMR studies

**Fig. 2:**
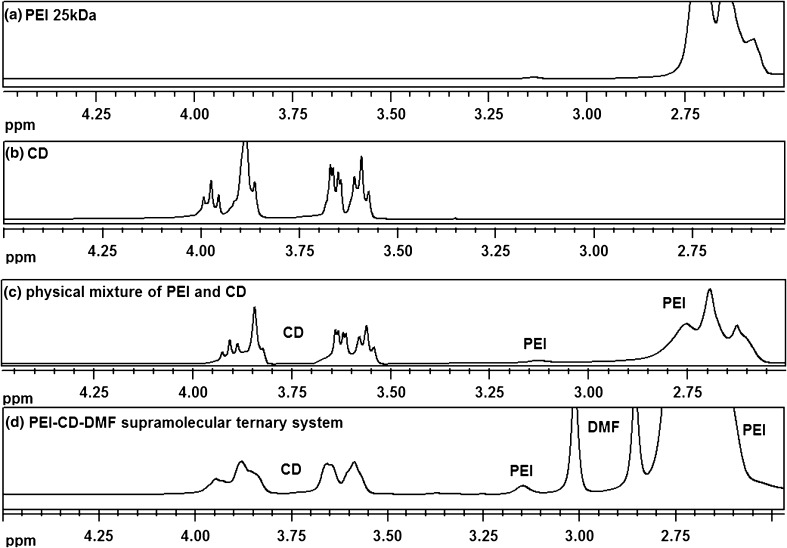
^1^H NMR spectrum (500 MHz, D_2_O) of PEI-βCD-DMF system (**d**), and its comparison with PEI 25 kDa (**a**), native CD (**b**), and physical mixture of PEI and CD (**c**)

Firstly, ^1^H and ^13^C spectra (in D_2_O) of the obtained systems and the starting reactants, were acquired. The differences in the ^1^H NMR spectrum of representative PEI-βCD-DMF (d) ternary system in comparison with: (a) pristine branched PEI 25 kDa, (b) native βCD, and (c) physical mixture of PEI and βCD, are presented in Fig. 2. The spectra of the products (Figs. S11-S18 in Supplementary Data) consist of peaks corresponding to the protons of PEI, CD and solvent (DMF or Py), but there are some differences in comparison with the spectra of the starting reactants (Figs. S1–S8 in Supplementary Data). The broadening of the signals coming from the CD might be attributed to the hydrogen bonding interactions between the CD and PEI or is associated with the formation of aggregates. It is worth mentioning that in the spectra of the βCD-solvent inclusion complexes (Figs. S9–S10 in Supplementary Data) and the βCD-PEI physical mixture in D_2_O (Fig. S18 in Supplementary Data), no peak broadening was observed. It is crucial to bear in mind that CD and PEI form hydrogen bonds also with water molecules, and those interactions crucially affect the arrangement of those compounds. If so it can be stated that the CD and branched PEI are not able enough to form stabile aggregates in the absence of the polar aprotic solvent, like DMF or Py, which also forms hydrogen bonds with the major building elements (PEI and CD). Please note that despite the fact that the products have been lyophilized for 24 h, the signals coming from the solvent molecules can be found in the spectra of each product.

**Fig. 3:**
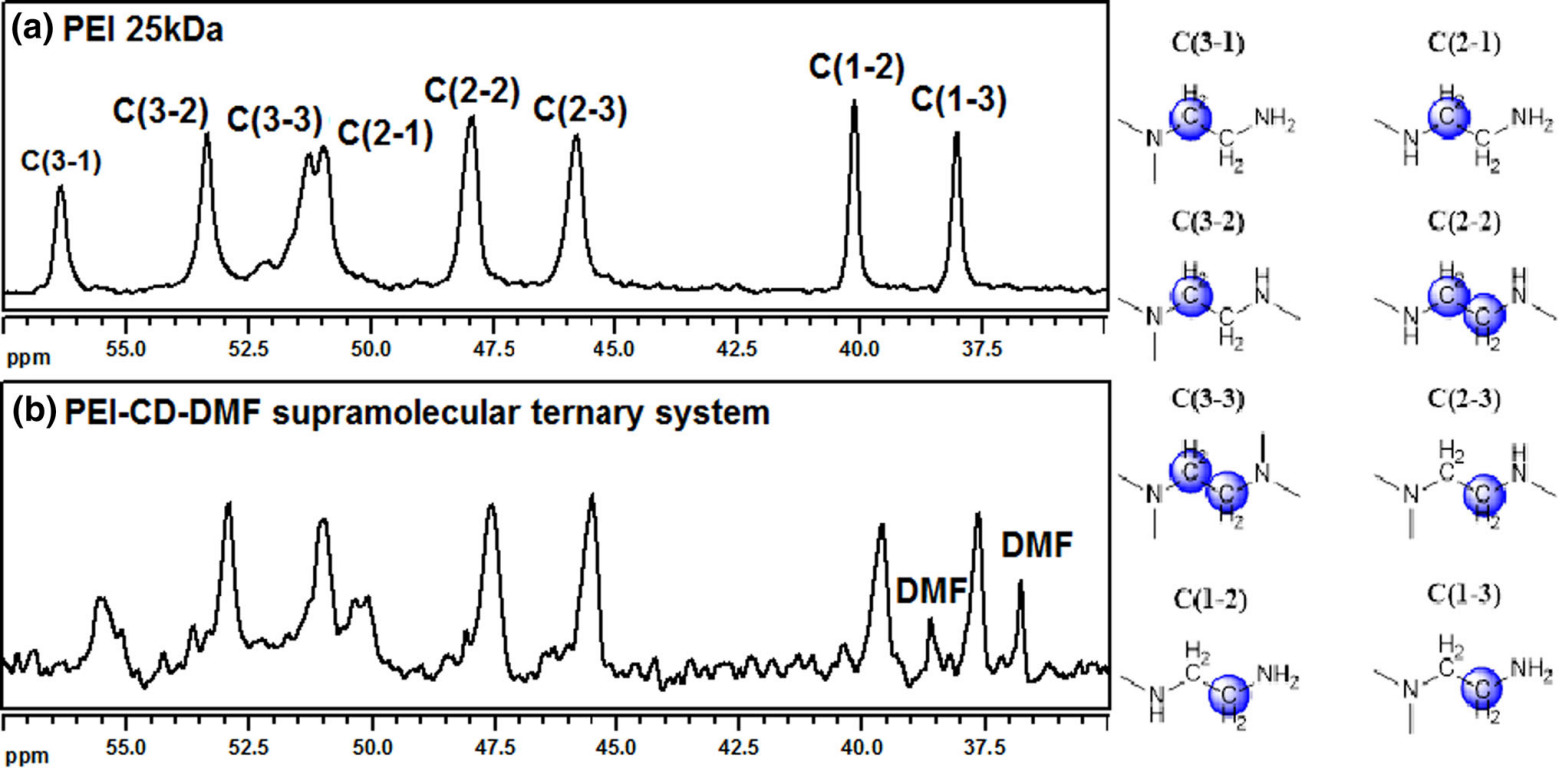
^13^C NMR spectrum (125 MHz, D_2_O) of PEI-βCD-DMF system (**b**), and its comparison with PEI 25 kDa (**a**) (designations are presented on the *right*)

Additionally, the specific changes were observed in the ^13^C NMR spectra of the representative PEI-CD-DMF systems (see Figs. S12, S14, S16 in Supplementary Data). These discussed differences (presented in Fig. 3) are related to the PEI broad peaks from the range ca. 50–52 ppm, which correspond to the carbons C(3–3) and C(2–1) (see the designations in Fig. 3) [30]. A direct comparison of the ^13^C NMR spectra of pristine PEI and PEI-βCD-DMF brings a conclusion that the peaks from C(3–3) and C(2–1) in the obtained supramolecular system appear as two individual and distinguished signals. This observation could be related to the hydrogen-bonding interactions between the tertiary amine moieties (HB acceptors) in the PEI structure and the hydrogen-donating functionalities (hydroxyl groups) in the CD molecules. Hence, we claim that the water-soluble solvent included in the ternary system structure (non-covalently bound to the branched PEI) allows the creation and the stabilization of aggregates through host-gest interactions with the CD ‘cup’.

### DOSY studies

It should be highlighted that a shifting of the signals coming from the protons of CD and guest molecules for ^1^H NMR spectra is very tiny. As for example for βCD complex with minoxidil the discussed differences are only ca. 0.005–0.08 ppm [31]. Therefore, such tiny shifting of the signals in the ^1^H NMR spectrum cannot be observed in the spectra of PEI-CD-solvent ternary systems because of the fact that the signals coming from CD are very broadened (please see Fig. 2).

The study of molecular diffusion in solution offers an insight into a range of physical molecular properties including molecular size, shape and aggregation states. The NMR-based measurements have been applied to many areas of chemistry for over five decades [32–34]. The mobility rates or diffusion coefficients may also be used as the basis for the separation of the spectra of compounds mixtures in the solution. This procedure being referred to as diffusion-ordered spectroscopy or DOSY. It is often regarded as a special chromatographic method for physical component separation, but unlike other techniques, it does not require any particular sample preparation or optimization of the chromatographic method.

All modern NMR-based diffusion measurements rely on the application of pulsed field gradients to map the physical location of a molecule in solution and have been made with conventional high-resolution NMR spectrometers through the provision of actively shielded pulsed field gradient (PFG) probeheads. Molecular diffusion is characterized along the direction of the applied field gradient, which is typically along the z-axis of conventional gradient probeheads. The measured signal is the integral over the whole sample volume and the NMR signal intensity is attenuated depending on the diffusion time and the gradient parameters. In practice, a series of NMR diffusion spectra are acquired as a function of the gradient strength. It can be observed that the intensities of the resonances follow an exponential decay. The rate of this decay depends on the diffusion coefficient. All signals corresponding to the same molecular species decay at the same rate. The next step is a transformation of raw PFG-NMR spectra into 2D-DOSY spectrum. The horizontal axis of the DOSY spectrum encodes the chemical shift of the observed nucleus (^1^H [ppm]). The vertical dimension encodes the diffusion constant (D [m^2^ s^−1^]). It is worth noticing that in the ideal case of non-overlapping component lines and no chemical exchange, the 2D peaks are aligned to horizontal lines, each corresponding to one sample of component–molecule.

Any non-covalent interaction, including host guest phenomena, between cyclodextrin and complexed guest molecules and/or polymer should be followed by reduced number in diffusion coefficients of the molecules included in the constructs in comparison with the pristine reactants [35–38]. In other words, if the molecules participate as separate compounds in given media, no changes in diffusion coefficients in comparison with starting values, are observed. It is also crucial to bear in mind that the amount of the molecule introduced into the solution affects the viscosity of a given solvent, thus, measured diffusion coefficient of the compound. Therefore, the observed value should be corrected by using an appropriate equation.

Further spectroscopic insight into the behavior of the obtained non-covalent systems in aqueous media, was based on above-mentioned diffusion-ordered NMR spectroscopy (DOSY). In the presented studies, diffusion coefficient values for each compound (CD, DMF/Py, and PEI) were calculated as follows: *D*_cor_ = *D*_meas_ × (*D*_HOD(ref)_ × *D*_HOD(obs)_^−1^), where *D*_cor_ is a viscosity-corrected diffusion coefficient value for each compound, *D*_meas_ stands for measured diffusion coefficient for each compound, *D*_HOD(ref)_ stands for diffusion coefficient for pristine D_2_O in pure solution, and *D*_HOD(obs)_ is an observed diffusion coefficient for the solvent with introduced molecules [39, 40]. The *D*_HOD(ref)_ value was measured to be 13.400 × 10^−10^ m^2^ s^−1^ (for DOSY spectrum of D_2_O, please see Fig. S19 in Supplementary Data). *D*_cor_ values for samples of pristine substrates were found to be 0.457 × 10^−10^, 1.958 × 10^−10^, 6.876 × 10^−10^, and 6.784 × 10^−10^ m^2^ s^−1^, for PEI 25 kDa, βCD, pyridine, and DMF, respectively (for DOSY spectra of the substrates, please see Figs. S20–S23 in Supplementary Data).

**Fig. 4:**
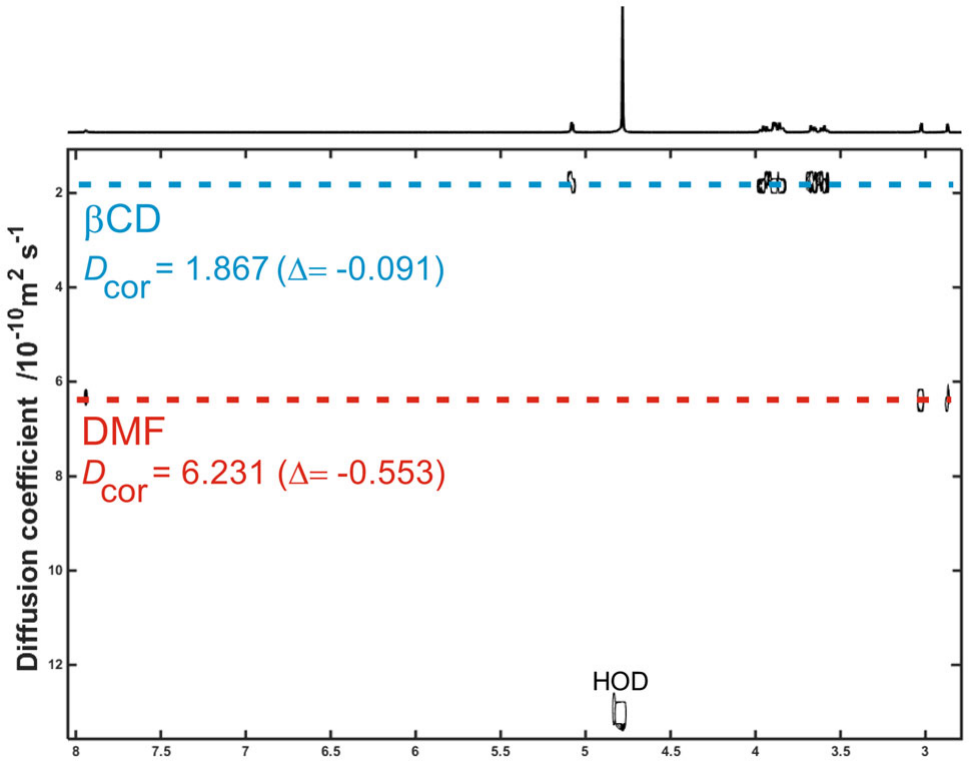
DOSY spectrum (500 MHz, D_2_O) of βCD-DMF inclusion complex with marked difference between calculated *D*_cor_ values and the numbers for corresponding substrates

**Fig. 5:**
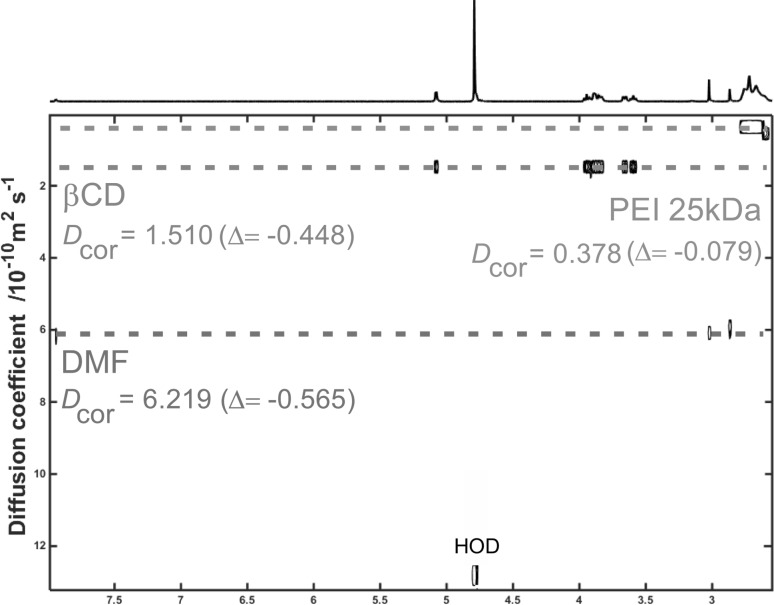
DOSY spectrum (500 MHz, D_2_O) of PEI-βCD-DMF non-covalent ternary system with marked difference between calculated *D*_cor_ values and the numbers for corresponding substrates

The DOSY spectra of the representative βCD-DMF complex and PEI-βCD-DMF ternary system are presented in Figs. 4 and 5, respectively. The DOSY spectra of βCD-Py inclusion complex and PEI-βCD non-covalent system with pyridine are shown in Figs. S24 and S25 in Supplementary Data. The *D*_cor_ values for each molecule included in the given system are summarized in Table 2. As it can be seen, for βCD-DMF complex (Fig. 4) the diffusion coefficients for both CD and DMF was found to be lower in comparison with the pristine reactants, which is a further indication of the formation of the inclusion complex between solvent (guest) molecule and cyclodextrin. Such phenomenon has been also observed both for βCD-Py complex (Fig. S24 in Supplementary Data) and PEI-βCD-solvent ternary systems (Fig. S25 in Supplementary Data). In the presented spectra of PEI-βCD-DMF system (Fig. 5) the diffusion coefficient values for each integrating element were found to be lower in comparison with the substrates. If so, one can conclude that PEI, CD and solvent molecules form non-covalent constructs. No differences in *D*_cor_ values in comparison with starting reactants (i.e. *D*_cor_ values for native: PEI, βCD and DMF) would be observed if supramolecular ternary system were not be formed. Please note that *D*_cor_ values for CD and solvent molecules are slightly lower than the diffusion coefficients observed in the corresponding CD-solvent inclusion complex. The lowering of *D*_cor_ value for each participating molecule can be attributed to the fact of shifting the equilibrium to form stable PEI-βCD-solvent aggregates in given media in the way of intermolecular non-covalent interactions between all the building elements. PEI with a molecular weight of 25 kDa is a large molecule in comparison with other integrating elements (CD, solvent), therefore, when the stabile systems are formed *D*_cor_ values for cyclodextrin and solvent (guest) are reduced.

**Table 2:** Diffusion coefficients (10^−10^ m^2^·s^−1^) of pristine substrates, inclusion complexes and ternary systems (*D*_HOD(ref)_ = 13.400, *D*_cor(PEI**)**_ **=** 0.457, *D*_cor(_β_CD**)**_ **=** 1.958, *D*_cor(Py**)**_ **=** 6.876, *D*_cor(DMF**)**_ **=** 6.784)

Molecule	*D*_meas_	*D*_HOD(obs)_	*D*_cor_	Difference to *D*_cor_ value for corresponding substrate
***Inclusion complex***				
*βCD-Py*				
Py	6.237	13.200	6.332	−0.554
βCD	1.751		1.778	−0.180
*βCD-DMF*				
DMF	6.161	13.250	6.231	−0.553
βCD	1.848		1.867	−0.091
***Ternary system***				
*PEI-βCD-Py*				
Py	6.026	13.070	6.117	−0.759
βCD	1.452		1.490	−0.468
PEI	0.323		0.342	−0.134
*PEI-βCD-DMF*				
DMF	6.085	13.090	6.219	−0.565
βCD	1.475		1.510	−0.448
PEI	0.369		0.378	−0.079
*PEI-βCD-DMF after 4 months*				
DMF	6.408	12.820	6.700	−0.084
βCD	1.633		1.707	−0.251
PEI	0.377		0.394	−0.063

DOSY spectrum of PEI-βCD-DMF sample after incubation in D_2_O at room temperature for 4 months, was also recorded (see Fig. S26 in Supplementary Data). The diffusion coefficients for each molecule forming the system were increased in comparison with starting PEI-βCD-DMF *D*_cor_ values (please see data presented in Table 2). This observation constitutes the confirmation of a well-known fact that the water molecules interfere the formation and strength of hydrogen bonding and host–guest type interactions. This feature of CD complexes and/or hybrids with polymers is a starting key-element for the design of the aforementioned controlled molecule (e.g. drug) release systems.

## 2D-ROESY NMR experiment

**Fig. 6:**
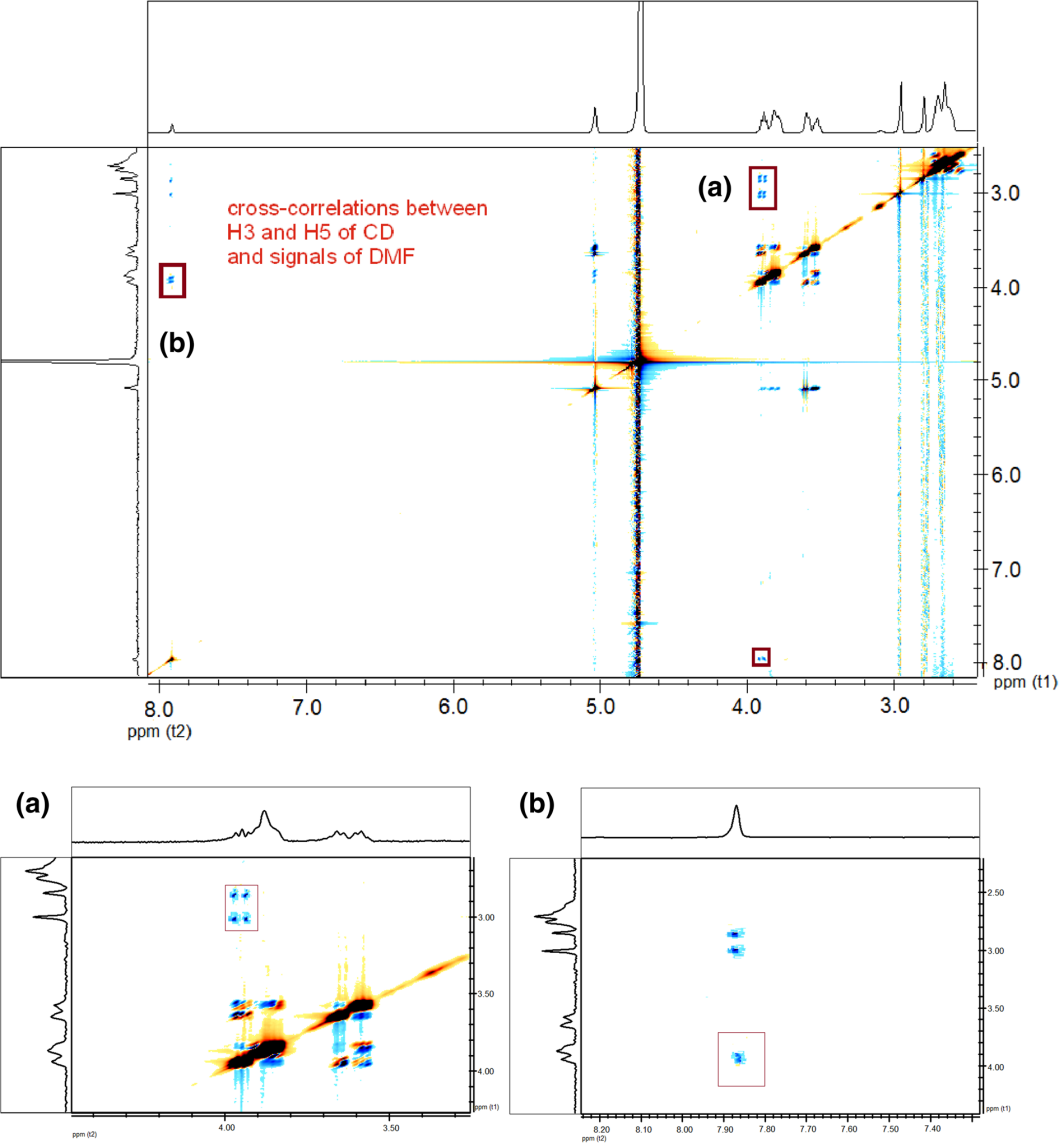
2D-ROESY NMR spectrum (500 MHz, D_2_O) of PEI-βCD-DMF non-covalent ternary system with marked crucial cross-correlations. Below full spectrum zooms that present the interesting parts of the spectrum are presented: (**a**) correlation between cyclodextrin H3 and H5 protons and aliphatic protons of DMF, (**b**) correlation between DMF C-H proton and cyclodextrin H3 and H5 protons

In order to unambiguously prove the host–guest interaction between the solvent molecules and CD in the obtained ternary systems, 2D-ROESY NMR spectrum in D_2_O was acquired. As presented in Fig. 6, the representative PEI-βCD-DMF system showed the cross-correlations between the signals of DMF (7.95, 3.03 and 2.88 ppm) and inner H3 and H5 protons of βCD (3.96–3.80 ppm). Please also note that same type of cross-correlations were found in the 2D-ROESY NMR spectrum in D_2_O of PEI-βCD-Py system (between Py aromatic protons and H3 and H5 protons of βCD; please see Fig. S27 in in Supplementary Data). This observation proves our assumption on the inclusion of the solvent molecules into the cavity of CD in D_2_O in the ternary system. It should be highlighted that correlations between PEI and CD cannot be observed due to a fact that using D_2_O as a solvent only CH_2_ groups of PEI can be found in the spectra. No correlation between NH_2_ of PEI and CD cavity can be observed due to a fast exchange of NH_2_ protons. Therefore, the distance of CH_2_ moieties of PEI to protons of CD is grater that 5 Å. In other words, the CD cavity is occupied only by guest (solvent) molecules not PEI, therefore PEI-CD cross correlations are not observed in the 2D-ROESY NMR spectrum.

### FTIR studies

More structural details were obtained by FT-IR spectroscopy. Please note that FT-IR studies allow to analyze the obtained materials in the solid state, and so, no ‘foreign’ solvent molecules (e.g. D_2_O) interfere with the analyzed supramolecular systems. Therefore, the infrared spectroscopy could be regarded as a key analysis to determine whether the non-covalent interactions take place in the obtained supramolecular systems. Such implementation of infrared spectroscopy in order to decipher and to confirm the occurrence of intermolecular interactions between the cyclodextrin and guest molecules (including linear polymers), has been previously demonstrated [19, 41]. Please note that all the obtained materials (i.e. ternary systems and CD complexes) were lyophilized for 24 h before acquiring FTIR spectra. On the other hand, native cyclodextrins and PEI were not lyophilized and both DMF and pyridine was not used as anhydrous solvent, therefore the FTIR spectra of the starting reactants include absorption bands coming from water molecules.

**Fig. 7:**
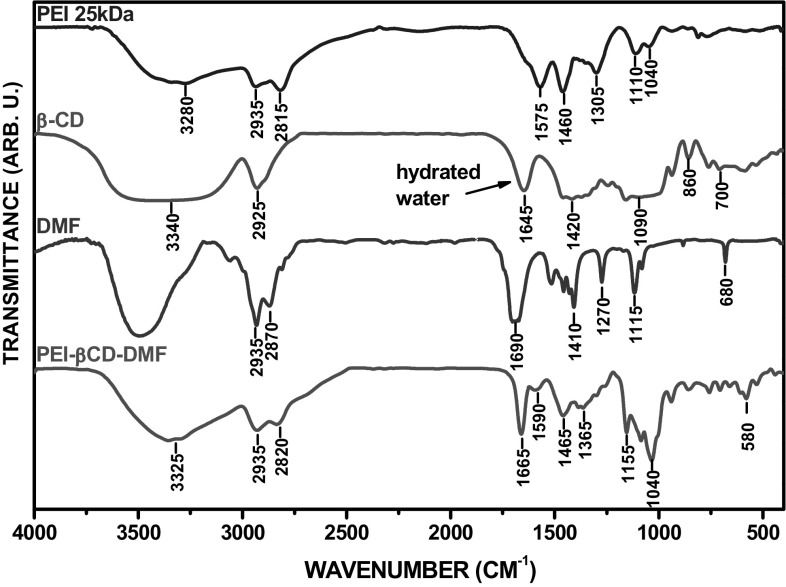
FT-IR spectra of the PEI-βCD-DMF system

The FT-IR spectrum of the representative PEI-βCD-DMF system is shown in Fig. 7. In the presented spectrum the characteristic bands coming from the starting reactants are seen, but there are some significant shifts of the absorption bands. Such observation is undoubtedly associated with the hydrogen-bonding/host–guest type interactions. As for example the sharp absorption band from the C=O moiety in DMF is downshifted of 25 cm^−1^ (from 1690 to 1665 cm^−1^), such as the weak absorption band associated with the amide band of DMF (580 cm^−1^; the downshift of 100 cm^−1^). Both observations constitute an irrefutable evidence of the inherent inclusion of the solvent molecules in the formation of this non-covalent ternary system. Such band-shifting of the absorption bands coming from the DMF moieties is also observed in the spectrum of the βCD-DMF inclusion complex (Fig. S28 in Supplementary Data). It means that the host–guest type interactions between the solvent molecules and CD may be a major factor that crucially influences the observed changes between the spectra of the obtained supramolecular systems and the starting reactants. Please note that the band located at 1590 cm^−1^ (Fig. 7), coming from the N–H vibrations of 1° amine moieties of PEI, is upshifted (pristine PEI: 1575 cm^−1^; the upshift of 15 cm^−1^). This finding represents a further confirmation of our statement about the non-covalent interactions between NH_2_ functionalities of PEI and the other structural elements of the aggregates. Such shifting of the C=O (1665–1630 cm^−1^) absorption bands coming from the solvent (DMF) and the primary amine groups of the branched PEI (1595–1580 cm^−1^) is also observed in the spectra of other ternary systems (Figs. S29–S30 in Supplementary Data). It is worth mentioning that in the PEI-βCD-Py spectrum (Fig. S31 in Supplementary Data) the main determinants of the non-covalent supramolecular interactions between the aggregates components are: (1) the lack of the absorption bands coming from pyridine: C–H stretching vibrations (3085–3005 cm^−1^), C=C and C–N ring bending vibrations (1490–1455 cm^−1^) and out of plane C–H bending vibrations (755–616 cm^−1^), and (2) the aforementioned PEI (NH_2_) absorption band are upshifted.

### Thermal stability of the obtained systems

According to TGA measurements, water content in the representative PEI-βCD-DMF and PEI-βCD-Py system was found to be ca. 1.7 and 1.5%, respectively (for TGA curves please see Figs. S32 and S33 in Supplementary Data). Water molecules interfere into the strength of the intermolecular non-covalent interactions, because of the hydrogen-bonding phenomena between hydroxyl groups (H_2_O) and other functionalities (e.g. included in the polymer structure). Hence, every loss of water molecules from the supramolecular structure should induce the enhanced strength of hydrogen bonding between the systems building elements. Therefore, we decided to try answer the question on the role of water molecules for the physicochemical features of the PEI-CD-solvent materials and the thermal stability of the obtained non-covalent systems.

It was found that the representative PEI-βCD-DMF system after drying for 24 h at 115 °C changes its form of a white viscous aggregate into the hard, bright-yellow solid. Moreover, the water solubility depletion of the obtained material was observed in comparison with starting structure, which is most probably associated with the strength of the hydrogen bonding network between PEI, CD and DMF. Such observation constitutes a strong evidence of the importance of water molecules on the stability of non-covalent systems. Therefore, we have confirmed the previously reported statements (e.g. Wang et al. [42]) that the role of water as a plasticizer is crucial for the lack of macroscopic glassy-like form of the obtained supramolecular PEI-CD systems.

In order to investigate the thermal stability of the obtained materials, βCD-DMF/Py complexes and PEI-βCD-DMF/Py ternary systems were heated under reduced pressure (2 mbar) at 200 °C for 24 h. As expected, the PEI-based materials changed their form into hard, bright-yellow solids and the water solubility was reduced in comparison with starting material. Such observation is correlated with the aforementioned loss of water after heating at 115 °C. Interestingly, ^1^H NMR spectra of the obtained materials after heating at 200 °C revealed that the solvent molecules are still present in the structures (see Figs. S34 and S35 in Supplementary Data). This phenomenon means that the non-covalent interactions between all the building elements in the obtained systems decrease the evaporation rate of the solvent which is included in the structure. TGA curves of the representative PEI-βCD-DMF and PEI-βCD-Py samples (Figs. S32 and S33 in Supplementary Data) constitute as a further confirmation of our observation, because of the fact that the evaporation of the solvent included in the ternary system structure begins at a higher temperature than for the pristine solvent. It other words, in the solid state the solvent molecules must exceed the energy barrier required to break the non-covalent bonds, which affects the decreased evaporation rate of the DMF or Py. Such enhanced thermal stability of CD inclusion complexes has been also observed for typically hydrophobic guest molecules complexed inside CD cavity [43].

### SEM and DLS analyses

**Fig. 8:**
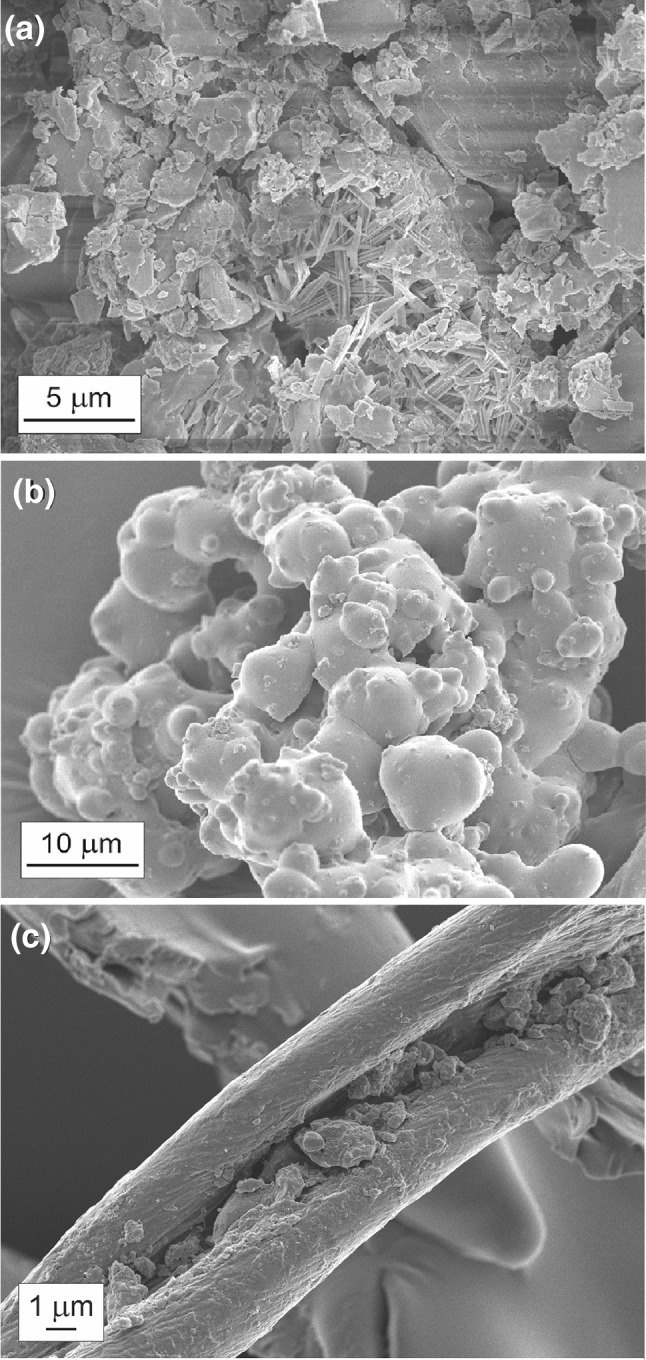
Representative SEM images of: (**a**) βCD-DMF complex, (**b**) PEI-βCD-DMF system and (**c**) PEI-βCD-DMF system after heating at 200 °C

The morphological details of the complexes in the solid state were obtained using scanning electron microscope. The representative microscopic images of the βCD-DMF complex (a), PEI-βCD-DMF supramolecular system (b) and PEI-βCD-DMF supramolecular system after heating at 200 °C for 24 h (c) are shown in Fig. 8. The changes in the morphology between the inclusion complex (without the polymer) and the aggregates (the ternary systems) are clearly seen. The βCD-DMF complex comprises of flake-like objects with the size between 5 and 20 μm. Moreover, these materials also contain some amount of rod-like structures. The PEI-βCD-DMF non-covalent supramolecular system is built of quasi-spherical micro-aggregates (diameter ca. 2–10 μm). Interestingly, PEI-βCD-DMF construct after heating in 200 °C for 24 h (Fig. 8c), comprises of some fiber-like objects with significant amount of opened “peapod” structures (width of ca. 10 μm).

Finally, the average hydrodynamic diameter in aqueous media (which corresponds to the particle size in the suspension) was determined by dynamic light scattering (DLS). The average diameter of the representative supramolecular systems comprising the βCD was found to be 465 and 510 nm, for PEI-βCD-Py and PEI-βCD-DMF, respectively (sample concentration 100 μg mL^−1^). Please note that those values are much higher in comparison with the pristine materials (CD: 90 nm [44] and PEI: 239 nm [45]).

## Conclusions

In conclusion, the efficient and easy to perform synthesis of the novel ternary supramolecular systems, comprising of the branched PEI, CD and the appropriate solvent molecules (DMF, pyridine), has been presented. The creation of the system is based on the hydrogen bonding interactions and host–guest phenomena. On the basis of a detailed study by means of the spectroscopic methods, the formation of supramolecular system, has been proposed. It has been found that the solvent molecules can be regarded as crucial integrating elements of the presented systems, allowing to form stable aggregates in given media. ROESY NMR spectra finally revealed that the solvent molecules form inclusion complexes with CD in the obtained ternary systems. By applying DOSY technique we have presented the in-depth insight into the host–guest chemistry and hydrogen-bonding dependent interactions of CD and the hydrophilic branched polymers. All the integrating elements of the constructs constitute as water-soluble molecules, but in aqueous media stabile aggregates between branched PEI, CD and DMF or Py, are formed. Therefore, our study presents the evidence for the universality and significance of DOSY technique for the analysis of the cyclodextrin complexes and its non-covalent systems with branched hydrophilic polymers. The results obtained by means of the DOSY technique constitute as a novel method for the analyses of the supramolecular constructs. Sequentially, we have demonstrated the influence of water molecules on the physicochemical features of the obtained materials. It was also found that the solvent molecules involved in the host–guest complexes and ternary systems, are present in the structures even after heating at 200 °C for 24 h. This observation means that the solvent molecules included in the structure of the obtained materials exhibit enhanced thermal stability. The TGA measurements undeniably confirmed the phenomenon of the increased evaporation temperature of the solvent molecules included in the ternary system structure. The electron microscopy studies revealed that in the solid state the supramolecular aggregates are composed of a sphere-like polymer core bearing the CD and solvent molecules. After heating the material under reduced pressure (2 mbar) at 200 °C, fiber-like structures, including “peapod-like” objects, are obtained. The average particle size of the obtained systems (aqueous media) is much bigger in comparison with all the pristine reactants, which constitute the further confirmation of the hypothesis about the formation of stable supramolecular PEI-CD systems. Our study presents the importance of direct non-covalent interactions between the branched hydrophilic polymer, cyclodextrin and polar guest molecules. The obtained systems may open up new strategies for the development of the supramolecular constructs dedicated to e.g. new polymer materials or adsorption technologies. Additionally, the added value of this paper is to demonstrate the scientific community that the non-covalent interactions between branched PEI and CD may take place in the DMF or Py, i.e. the solvents commonly used as a reaction medium or the additive during a formation of covalent-type PEI-CD structures. It means that herein presented phenomena should be always taken into account during a synthesis of the polymer materials comprising CD, which are conducted in the solvents forming possible host–guest complexes with CD.

## Electronic supplementary material

Below is the link to the electronic supplementary material.

Supplementary material 1 (AVI 3483 kb)

Supplementary material 2 (DOC 3207 kb)
