# Supplementary material for: Novel non-covalent stable supramolecular ternary system comprising of cyclodextrin and branched polyethylenimine
Source: J Incl Phenom Macrocycl Chem. 2016 Nov 9;87(1):53–65. doi: 10.1007/s10847-016-0677-1 (PMC5310563; doi:10.1007/s10847-016-0677-1)
Supplement: Supplementary file 2 — Supplementary material 2 (DOC 3207 kb) [file 10847_2016_677_MOESM2_ESM.doc]

**Novel non-covalent stable supramolecular ternary system comprising of cyclodextrin and branched polyethylenimine**

Artur Kasprzak ^a^*, Magdalena Poplawska ^a^, Hanna Krawczyk ^a^, Sergey Molchanov ^a^, Mikolaj Kozlowski ^a^ and Michal Bystrzejewski ^b^

^a^ Faculty of Chemistry, Warsaw University of Technology, 00-664 Warsaw, Poland

^b^ Department of Chemistry, University of Warsaw, 02-093 Warsaw, Poland

**Supplementary Data**

Content list

[1. Solubility behavior of the obtained systems 2](#__RefHeading___Toc462912642)

[2. ^1^H and ^13^C NMR spectra 3](#__RefHeading___Toc462912643)

[2.1 ^1^H and ^13^C NMR spectra of the pristine CDs and PEI 25kDa 4](#__RefHeading___Toc462912644)

[2.2 ^1^H and ^13^C NMR spectra of the βCD-solvent complexes 8](#__RefHeading___Toc462912645)

[2.3 ^1^H and ^13^C NMR spectra of the obtained systems and PEI-βCD physical mixture 9](#__RefHeading___Toc462912646)

[3. DOSY spectra 13](#__RefHeading___Toc462912647)

[4. 2D ROESY NMR spectrum of PEI-βCD-Py supramolecular ternary system 17](#__RefHeading___Toc462912648)

[5. FT-IR spectra 18](#__RefHeading___Toc462912649)

[6. Thermal stability of the obtained systems 20](#__RefHeading___Toc462912650)

# Solubility behavior of the obtained systems

***Table S1****.* Solubility behavior of the pristine substrates, CD complexes and the obtained ternary systems a

| **Pristine substrates** | | | | |
| --- | --- | --- | --- | --- |
| Compound | Solubility | | | |
|  | **H_2_O** | **DMSO** | **MeOH** | **CHCl_3_** |
| **PEI 25kDa** | YES | NO | YES | YES |
| **CD (α,β,γ)** | YES | YES | NO | NO |
| **CD-solvent inclusion complexes** | | | | |
| Complex | Solubility | | | |
|  | **H_2_O** | **DMSO** | **MeOH** | **CHCl_3_** |
| **CD(αCD,β,γ)-DMF** | YES | YES | NO | NO |
| **βCD-Py** | YES | YES | NO | NO |
| **Ternary systems** | | | | |
| System | Solubility | | | |
|  | **H_2_O** | **DMSO** | **MeOH** | **CHCl_3_** |
| **PEI-CD(α,β,γ)-DMF** | YES | TURBID MIXTURE | NO | NO |
| **PEI-βCD-Py** | YES | SLIGHTLY TURBID MIXTURE | NO | NO |

^a^ probes concentration 400 μg/ml.

# ^1^H and ^13^C NMR spectra

The signals on the spectra were designated as it is presented below**:**

- For CDs:

- For PEI 25kDa:

## 2.1 ^1^H and ^13^C NMR spectra of the pristine CDs and PEI 25kDa

*Fig. S1.* ^1^H NMR spectrum (500 MHz, D_2_O) of αCD

*Fig. S2.* ^13^C NMR spectrum (125 MHz, D_2_O) of αCD

*Fig. S3.* ^1^H NMR spectrum (500 MHz, D_2_O) of βCD (see designations in Fig. S1)

*Fig. S4.* ^13^C NMR spectrum (125 MHz, D_2_O) of βCD (see designations in Fig. S2)

*Fig. S5.* ^1^H NMR spectrum (500 MHz, D_2_O) of γCD (see designations in Fig. S1)

*Fig. S6.* ^13^C NMR spectrum (125 MHz, D_2_O) of γCD (see designations in Fig. S2)

*Fig. S7.* ^1^H NMR spectrum (500 MHz, D_2_O) of PEI 25kDa

*Fig. S8.* ^13^C NMR spectrum (125 MHz, D_2_O) of PEI 25kDa

## 2.2 ^1^H and ^13^C NMR spectra of the βCD-solvent complexes

*Fig. S9.* ^1^H NMR spectrum (500 MHz, D_2_O) of βCD-DMF complex

***Fig. S10.*** ^1^H NMR spectrum (500 MHz, D_2_O) of βCD-Py complex

## 2.3 ^1^H and ^13^C NMR spectra of the obtained systems and PEI-βCD physical mixture

***Fig. S11.*** ^1^H NMR spectrum (500 MHz, D_2_O) of PEI-αCD-DMF system

***Fig. S12.*** ^13^C NMR spectrum (125 MHz, D_2_O) of PEI-αCD-DMF system

***Fig. S13.*** ^1^H NMR spectrum (500 MHz, D_2_O) of PEI-βCD-DMF system

***Fig. S14.*** ^13^C NMR spectrum (125 MHz, D_2_O) of PEI-βCD-DMF system (see designations in Fig. S12)

***Fig. S15.*** ^1^H NMR spectrum (500 MHz, D_2_O) of PEI-γCD-DMF system (see designations in Fig. S11)

***Fig. S16.*** ^13^C NMR spectrum (125 MHz, D_2_O) of PEI-γCD-DMF system (see designations in Fig. S12)

***Fig. S17.*** ^1^H NMR spectrum (500 MHz, D_2_O) of PEI-βCD-Py system

***Fig. S18.*** ^1^H NMR spectrum (500 MHz, D_2_O) of the PEI and βCD physical mixture in D_2_O

# DOSY spectra

*D*_HOD(obs)_ values (see equation in subsection 3.2 in the main text) were always measured for 4.79 ppm signal.

**
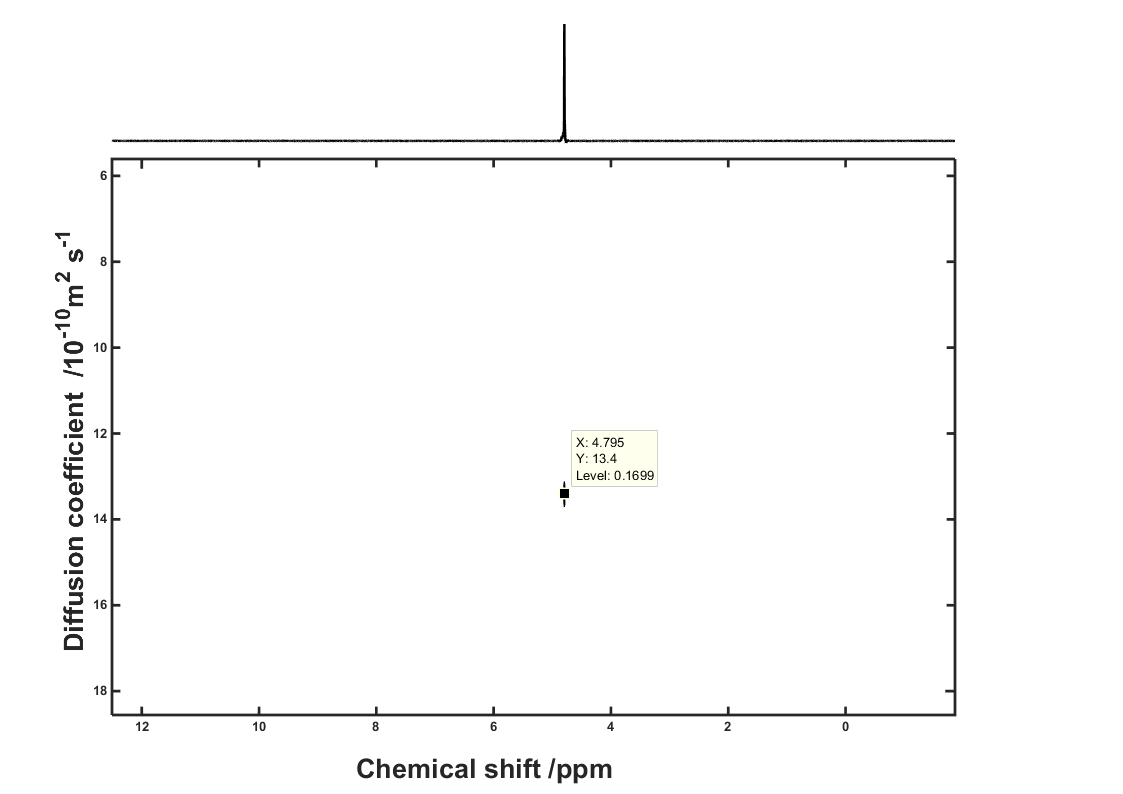
**

***Fig. S19.*** DOSY spectrum (500 MHz) of D_2_O

**
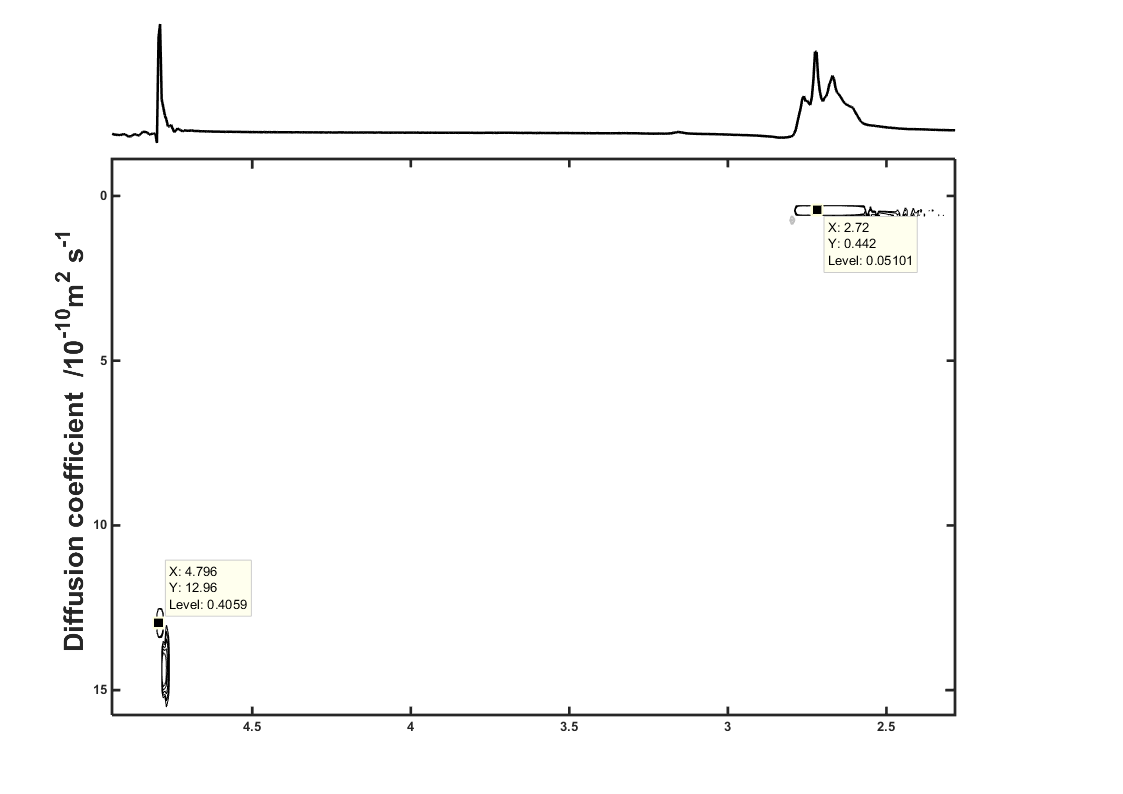
**

***Fig. S20.*** DOSY spectrum (500 MHz, D_2_O) of PEI 25kDa

**
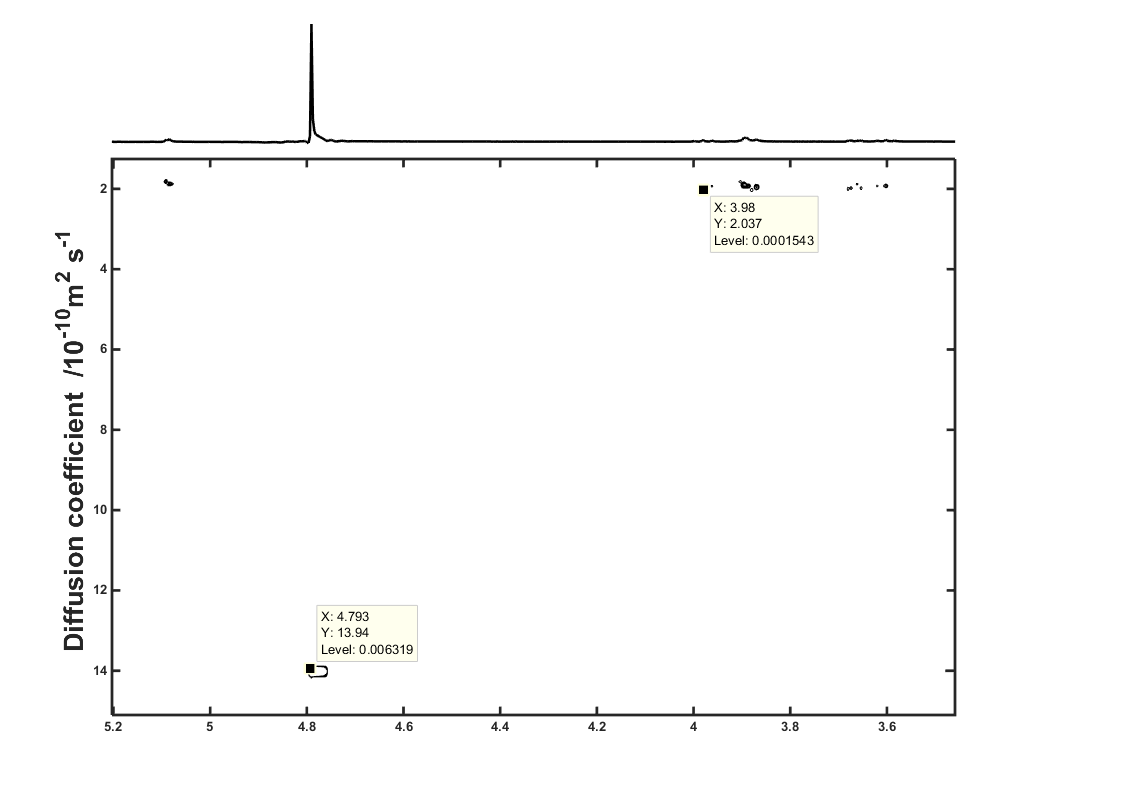
**

***Fig. S21.*** DOSY spectrum (500 MHz, D_2_O) of βCD

**
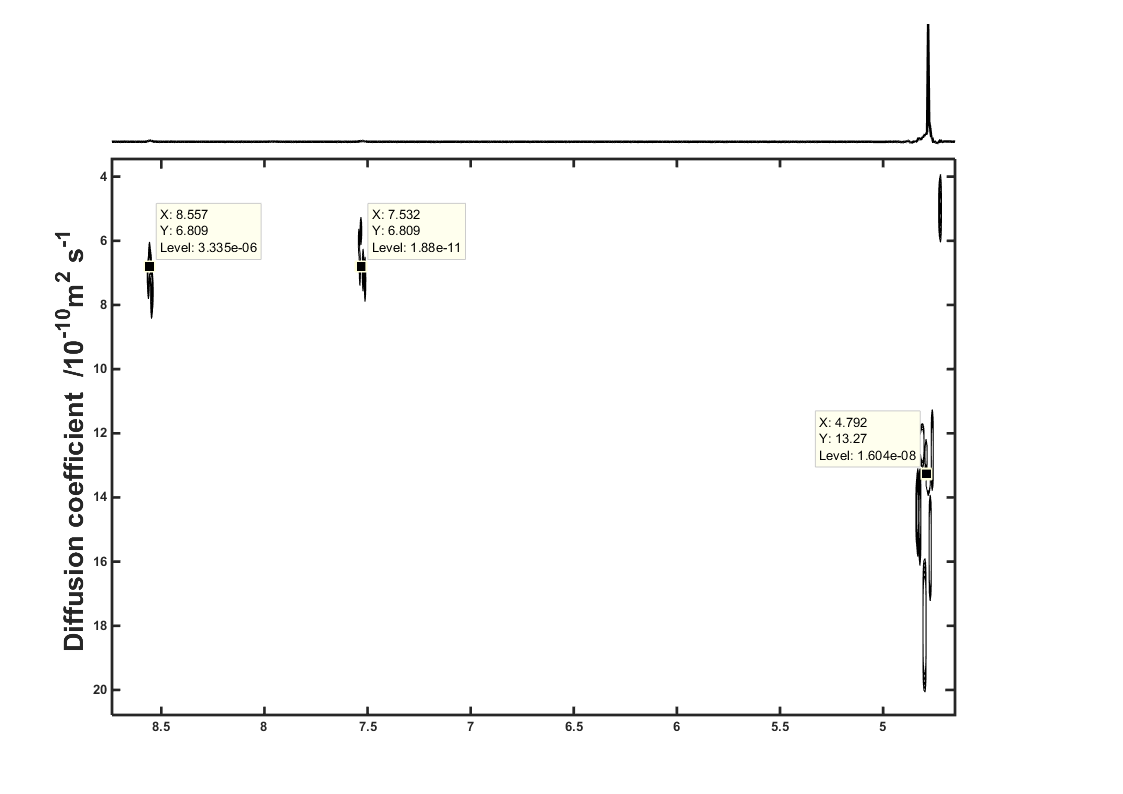
**

***Fig. S22.*** DOSY spectrum (500 MHz, D_2_O) of Py

**
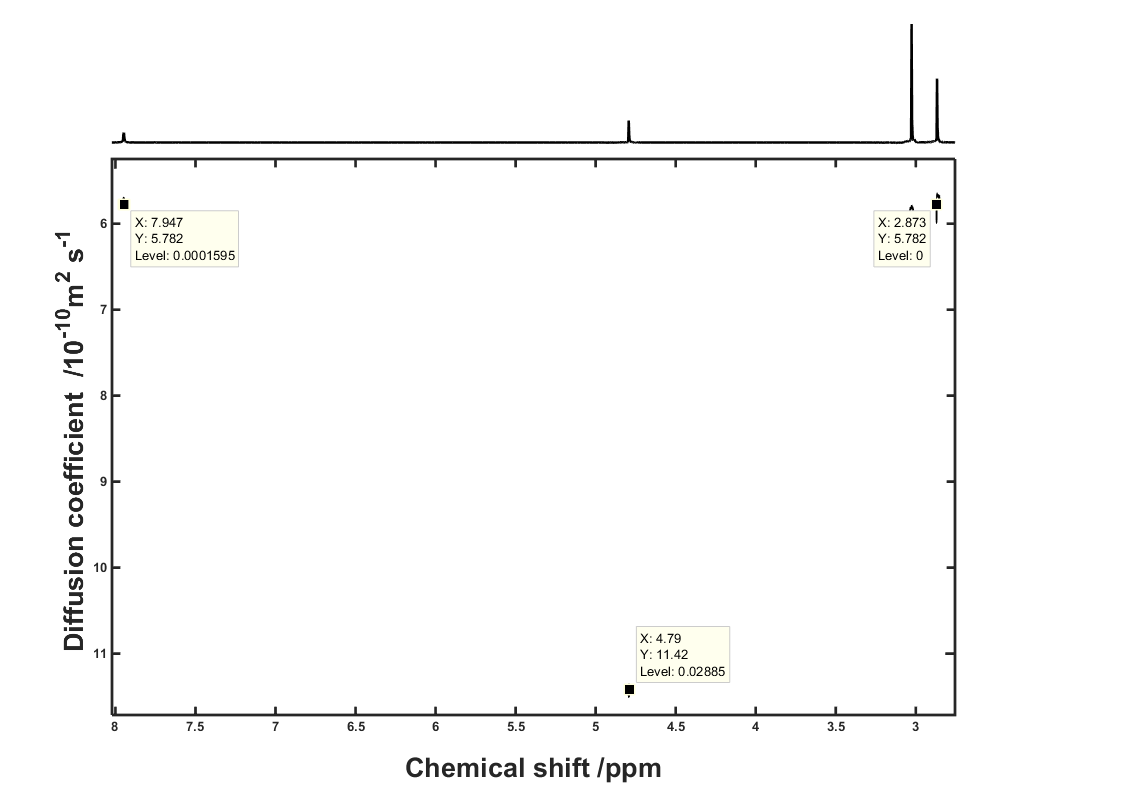
**

***Fig. S23.*** DOSY spectrum (500 MHz, D_2_O) of DMF

**
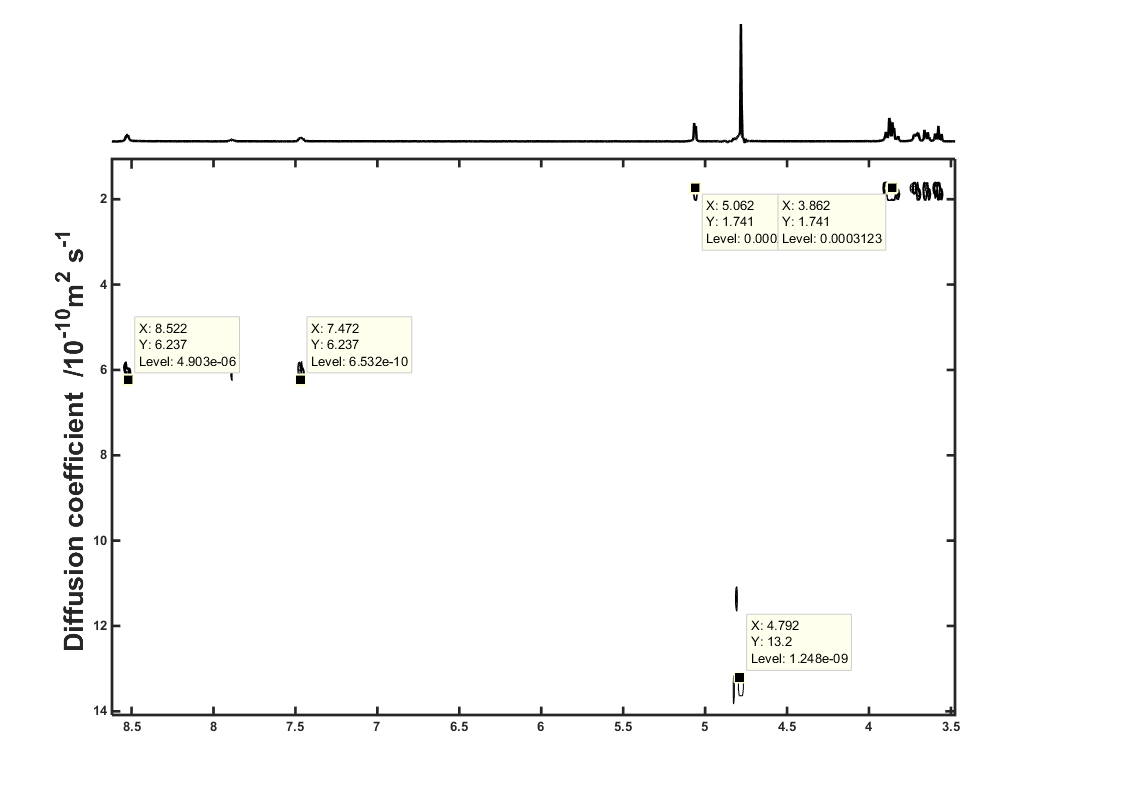
**

***Fig. S24.*** DOSY spectrum (500 MHz, D_2_O) of the βCD-Py inclusion complex in D_2_O

**
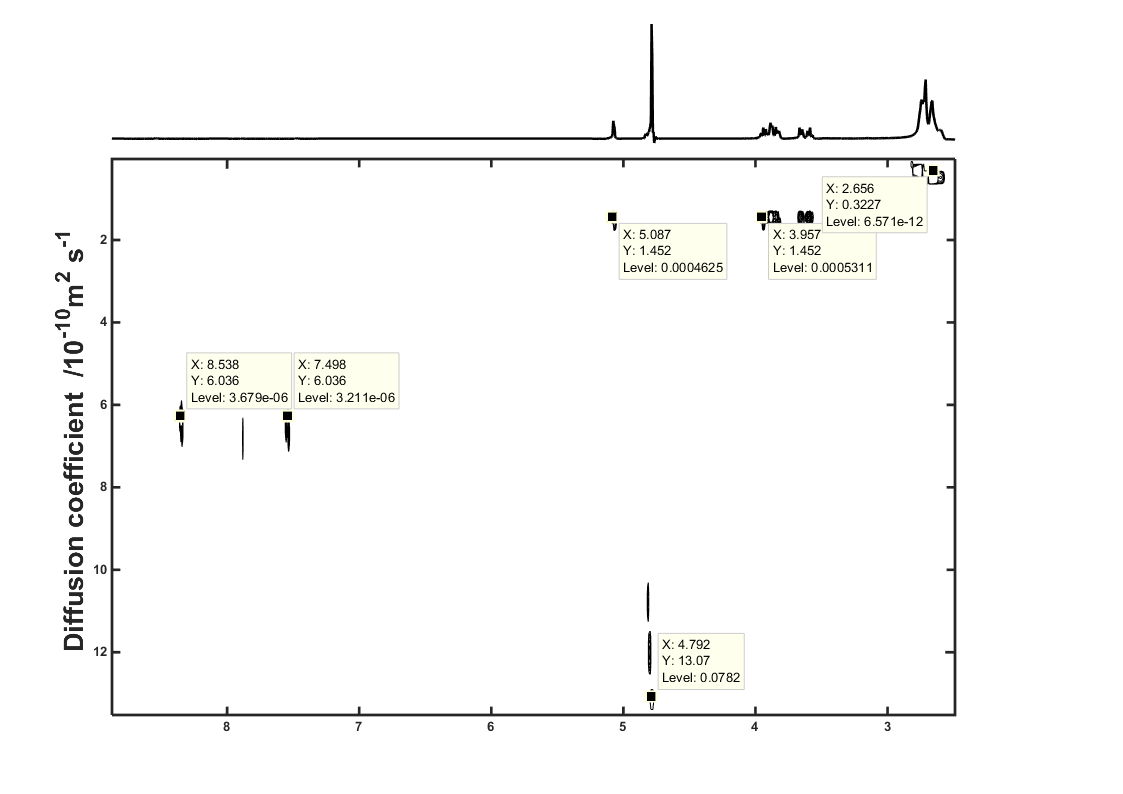
**

***Fig. S25.*** DOSY spectrum (500 MHz, D_2_O) of the PEI-βCD-Py ternary system in D_2_O

**
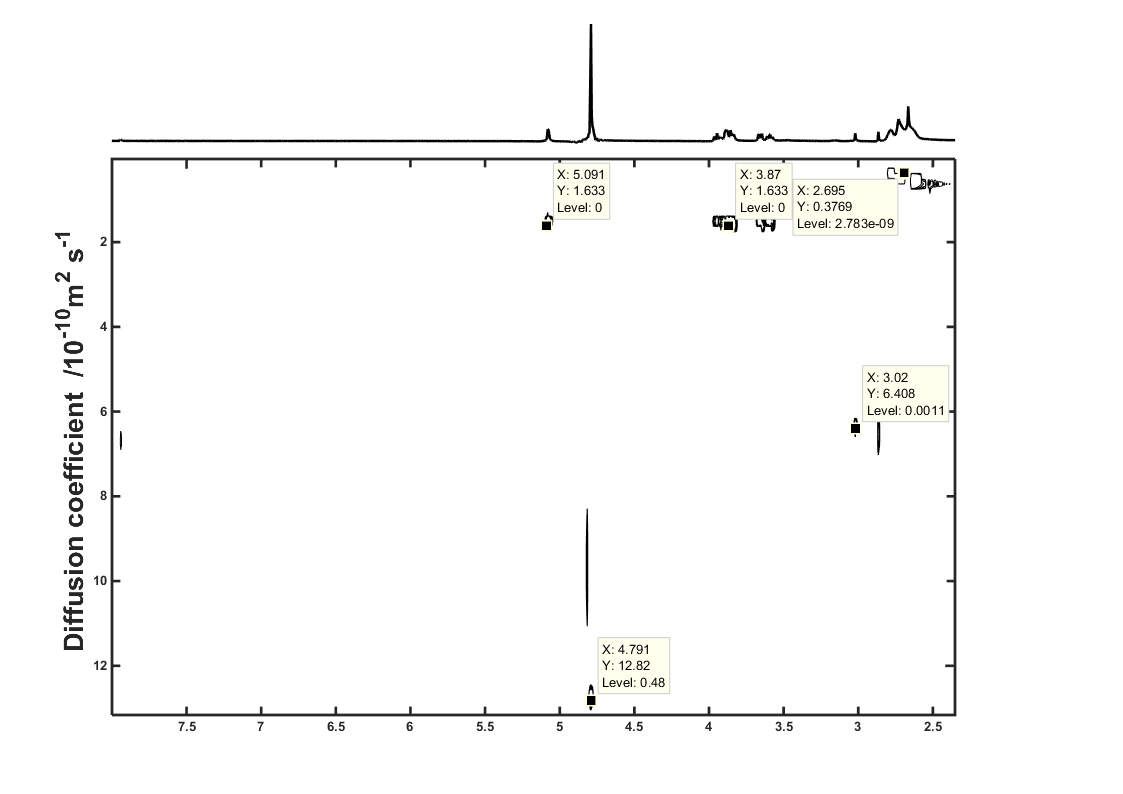
**

***Fig. S26.*** DOSY spectrum (500 MHz) of the PEI-βCD-DMF ternary system after 4 months of incubation in D_2_O

# 2D ROESY NMR spectrum of PEI-βCD-Py supramolecular ternary system


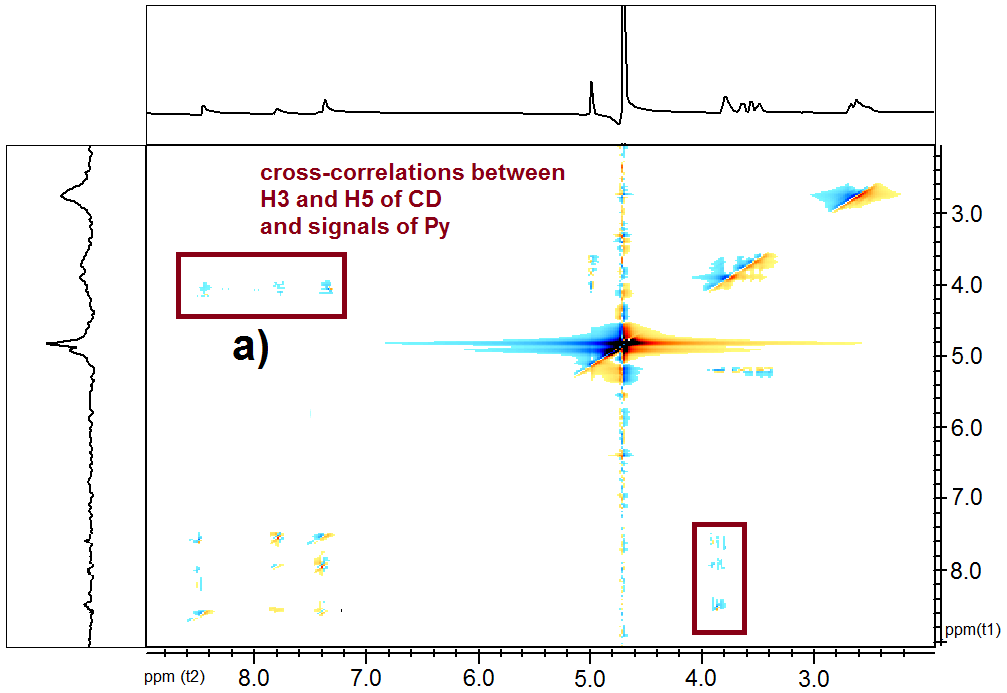


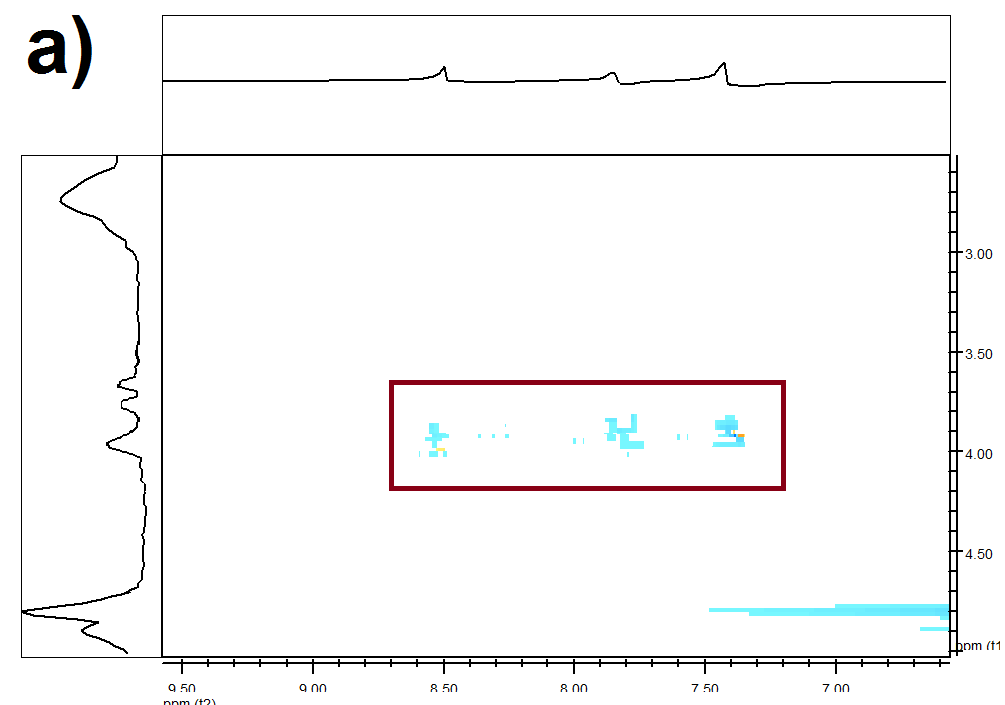


***Fig. S27*.** 2D-ROESY NMR spectrum (500 MHz, D_2_O) of PEI-βCD-Py non-covalent ternary system with marked crucial cross-correlations. Below full spectrum zoom that present the interesting part of the spectrum is presented: **a)** correlation between cyclodextrin H3 and H5 protons and aromatic protons of Py

# FT-IR spectra

***Fig. S28.*** FT-IR spectrum of βCD-DMF complex

***Fig. S29.*** FT-IR spectrum of PEI-αCD-DMF system

***Fig. S30.*** FT-IR spectrum of PEI-γCD-DMF system

***Fig. S31.*** FT-IR spectrum of PEI-βCD-Py system

# Thermal stability of the obtained systems

***Fig. S32.*** TGA curve (in nitrogen; with first derivative) of PEI-βCD-DMF system

***Fig. S33.*** TGA curve (in nitrogen; with first derivative) of PEI-βCD-Py system

***Fig. S34.*** ^1^H NMR spectrum (500 MHz, D_2_O) of βCD-DMF complex after heating under reduced pressure (2 mbar) at 200 °C for 24 h

***Fig. S35.*** ^1^H NMR spectrum (500 MHz, D_2_O) of PEI-βCD-DMF ternary system after heating under reduced pressure (2 mbar) at 200 °C for 24 h
